# Small Molecule Supplements Improve Cultured Megakaryocyte Polyploidization by Modulating Multiple Cell Cycle Regulators

**DOI:** 10.1155/2017/2320519

**Published:** 2017-10-19

**Authors:** Xiaojing Zou, Mingyi Qu, Fang Fang, Zeng Fan, Lin Chen, Wen Yue, Xiaoyan Xie, Xuetao Pei

**Affiliations:** ^1^Stem Cell and Regenerative Medicine Lab, Beijing Institute of Transfusion Medicine, Beijing 100850, China; ^2^South China Research Center for Stem Cell & Regenerative Medicine, Guangzhou 510005, China; ^3^Southern Medical University, Guangzhou 510515, China

## Abstract

Platelets (PLTs) are produced by megakaryocytes (MKs) that completed differentiation and endomitosis. Endomitosis is an important process in which the cell replicates its DNA without cytokinesis and develops highly polyploid MK. In this study, to gain a better PLTs production, four small molecules (Rho-Rock inhibitor (RRI), nicotinamide (NIC), Src inhibitor (SI), and Aurora B inhibitor (ABI)) and their combinations were surveyed as MK culture supplements for promoting polyploidization. Three leukemia cell lines as well as primary mononuclear cells were chosen in the function and mechanism studies of the small molecules. In an optimal culture method, cells were treated with different small molecules and their combinations. The impact of the small molecules on megakaryocytic surface marker expression, polyploidy, proliferation, and apoptosis was examined for the best MK polyploidization supplement. The elaborate analysis confirmed that the combination of SI and RRI together with our MK induction system might result in efficient ploidy promotion. Our experiments demonstrated that, besides direct downregulation on the expression of cytoskeleton protein actin, SI and RRI could significantly enhance the level of cyclins through the suppression of p53 and p21. The verified small molecule combination might be further used in the in vitro PLT manufacture and clinical applications.

## 1. Introduction

Platelets (PLTs) are a component of blood, which plays an important role in hemostasis, wound healing, inflammation, and thrombosis [1, 2]. Many diseases could cause thrombocytopenia, some of which caused by hematologic diseases, radiotherapy, and chemotherapy is difficult to restore by itself. Severe thrombocytopenia will result in spontaneous bleeding, which is seriously harmful to the life and health of patients. Repeated prophylactic platelet transfusions are required for the disease treatment. However, the clinical available platelets are far below the amount of demand. Fortunately, the current study showed that in vitro platelet generation from stem cells is an alternative way to get platelets for clinical usage [3–5].

Hematopoietic stem cells differentiate to megakaryocytes and release of platelets under the influence of the bone marrow microenvironment. During megakaryocyte differentiation and maturation, the cells would increase their DNA content, cytoplasm volume, and surface area of membrane. The large size and abundant cytoplasm eventually promote the release of a large number of platelets. In normal bone marrow, megakaryocytes form up to 128N polyploid compared to no more than 16N ploidy from in vitro cell culture, and most cells stayed at the 2N and 4N stage after tissue culture [6]. The obtainment of the high polyploid megakaryocytes, which produce significant amounts of platelets, will provide a new source for the clinical supplements of platelet transfusion.

Considering the importance of megakaryocyte polyploidy in platelet generation, exploring the mechanism under megakaryocyte polyploidization became one of the hotspots in the field. It is suggested that endomitosis without anaphase B, as well as the blockage of cytokinesis, results in megakaryocyte polyploidization. Four cytokinesis inhibitors, including Rho-Rock inhibitor (RRI, Y27632), nicotinamide (NIC, vitamin B3), Src inhibitor (SI, Su6656), and Aurora B kinase inhibitor (ABI, ZM447439), were chosen to increase the polyploidization output during megakaryocyte induction in vitro [7]. Although the study by Avanzi et al. suggested that RRI alone could produce a high final ploidy compared to other small molecules or their combinations, it is still unclear whether the protocol is ubiquitous to all the megakaryocytes, and the molecular mechanism underlined still remains unknown. Here, we took advantage of the stability of leukemia cell line megakaryocytic differentiation model to verify the best polyploidy induction supplements and tried to figure out relevant cell cycle regulators which might lay upstream of cytokinesis regulation.

## 2. Materials and Methods

### 2.1. Materials

The following regents were used: Annexin V-FITC/PI Apoptosis Detection Kit (Dojindo, Japan); phorbol 12-myristate 13-acetate (PMA), nicotinamide, and RPMI-1640 medium (Sigma America); Rho-Rock inhibitor (Stemgent America); Src inhibitor and Aurora B kinase inhibitor (Millipore America); PE-antiCD41 and APC-antiCD61 antibodies (eBioscience America); anti-p21, anti-p53, and anti-cyclin B1 antibodies (Santa Cruz, America); anti-*β*-actin and anti-GAPDH antibodies (Cell Signaling Technology America). All cytokines were purchased from Preprotech America.

### 2.2. Cell Culture

Mononuclear cells (MNCs) were isolated from fresh human umbilical cord blood after the delivery of normal pregnancies with patient's informed consent. MNCs were applied to density centrifugation at 700*g* for 25 min at room temperature using Lymphocytes Separation Medium in 1.077 g/mL (TBD sciences, China). MNCs (1 × 10^6^ cells/well) were cultured in StemSpan SFEM medium (StemCell Technologies, Canada) supplemented with SCF (50 ng/mL), TPO (100 ng/mL), IL-3 (20 ng/mL), IL-6 (50 ng/mL), and IL-11 (20 ng/mL). After 10 days culture, the cells expressed the MK markers CD41 and CD61 (Figure S1 in Supplementary Material available online at https://doi.org/10.1155/2017/2320519) and we refer to them as MK progenitors (MK-PROs).

The human leukemia K562, MEG-01, and UT-7 cells were cultured in RPMI-1640 medium supplemented with 10% FBS. Recombinant human erythropoietin (EPO, 1 IU/ml) was added to the medium for UT-7 cell maintenance. To induce megakaryocytic differentiation, cells were seeded at 2 × 10^5^ cells/ml and cultured and treated with PMA.

### 2.3. Flow Cytometric Analysis for MK Ploidy

Cells were collected and washed with PBS and then permeabilized with 70% cold methanol for 1 hour at 4°C or preserving at −20°C. Propidium iodide (50 *μ*g/ml, Sigma, America) and RNase (Promega, America) were added for DNA staining for 30 min and analysed by BD FACS Calibur.

### 2.4. Morphologic Analysis

Cells were rinsed in PBS, cytocentrifuged onto glass slides, stained with Wright–Giemsa solution (Sigma), and then observed by light microscopy (NIKON, Japan). NIS-Elements F software was used to capture picture and measure cell diameter. Over 50 cells from five random views were measured. Dunnett's *t*-tests were used for statistical analysis.

### 2.5. Cell Proliferation Assay

The K562 and MEG-01 cells were treated with PMA (0.625 ng/ml) for 3 days and then with PMA (10 ng/ml) with or without different small molecules. The cells were cultured by the concentration of 2 × 10^4^ in 96-well plate (100 *μ*l/well) and the cell activity was checked by adding CCK-8 solution according to the manufacturer's instruction (Dojindo, Japan).

### 2.6. Quantitative RT-PCR

Cells were lysed and total RNA was isolated by Trizol Reagent (Invitrogen, America), followed by reverse transcription of 1 *μ*g total RNA with the reverse transcriptase M-MLV (Takara, Japan) using oligo (dT) primers. Quantitative RT-PCR reactions were performed with SYBR Green real-time PCR Master Mix (TOYOBO, Japan) on Bio-Rad iQ5 System (Bio-Rad, USA). The amount of mRNA for each sample was normalized using GAPDH as endogenous control. The primer sequences for quantitative RT-PCR were listed in Table 1.

### 2.7. Statistical Analysis

All results were replicated in three independent experiments. All statistics data are expressed as the mean ± SD. Statistical analysis was performed using the SPSS software (ver. 18.0 for Windows; SPSS, Chicago, IL). The groups of the culture methods in polyploidization and effects of several small molecules were analyzed by using ANOVA pairwise comparison. Two-Way ANOVA and LSD-*t* Multiple Comparison Test for multiple comparisons were applied. *p* value of less than 0.05 was considered significant. ^*∗*^*p* < 0.05; ^*∗∗*^*p* < 0.01; ^*∗∗∗*^*p* < 0.001.

## 3. Results

### 3.1. Effect of Small Molecules on MK Polyploidization

**Figure 1:**
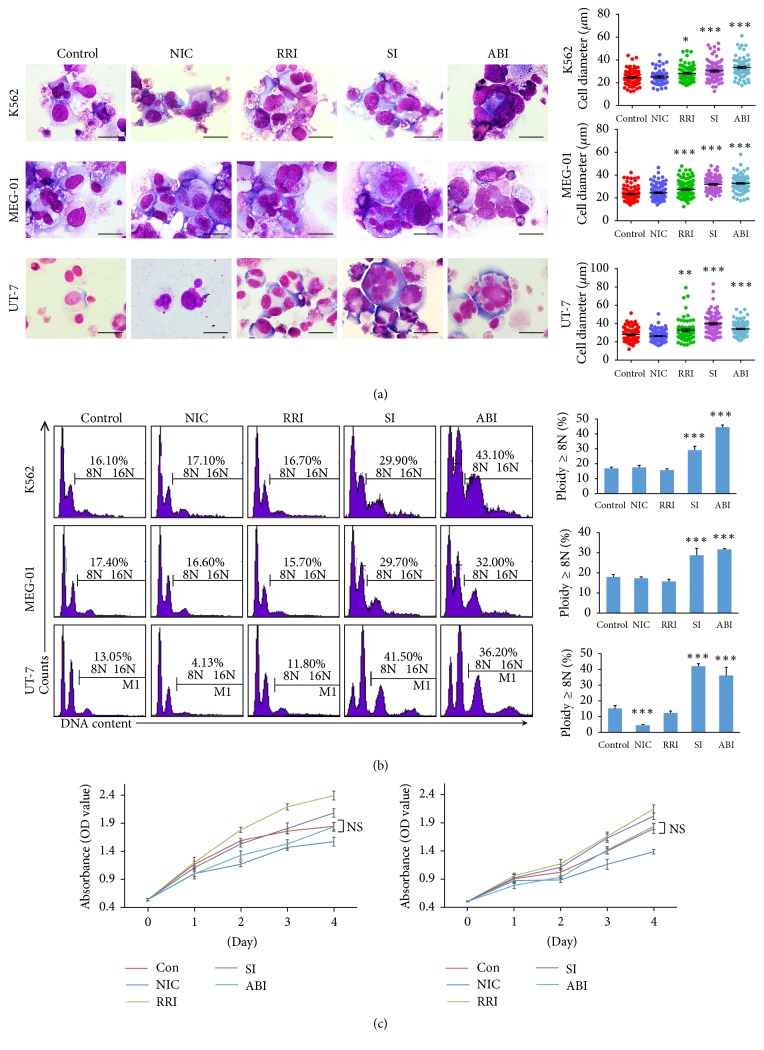
Effect of small molecules on MK polyploidization in megakaryocytic cells. (a) Morphological difference of the cells after treatment with small molecules was shown by Wright–Giemsa staining. Cell diameter of over 50 cells from five random views was measured (scale bars: 20 *μ*m). (b) DNA ploidy analysis by flow cytometry. Ploidy (≥8N) was compared with control in three cell lines. (c) The proliferation of K562 and MEG-01 cells with different small molecules evaluated by CCK8 assay. ^*∗*^*p* < 0.05; ^*∗∗*^*p* < 0.01; ^*∗∗∗*^*p* < 0.001.

PMA was commonly used in induction of megakaryocyte differentiation from human leukemic cells, such as K562, MEG-01, and UT-7 cells in this study. After PMA treatment, the cells became larger and polyploid and expressed the MK markers CD41 and CD61 (Figure S2). To determine whether the selected small molecules could further improve polyploidization, we added small molecules to the culture medium after MK differentiation, respectively (Figure S3). The increase in cell size and ploidy levels after RRI, ABI, or SI treatment was evident in cytospin analysis (Figure 1(a)). However, considering flow cytometry test, in all 3 cell lines, statistically significant difference in the percentage of highly polyploidy megakaryocytes was not detectable in RRI group compared with control group. Also, NIC had no effect in K562 and MEG-01 cells and even reduced the percentage in UT-7 cells. In contrast, addition of SI or ABI alone resulted in more percentage of highly polyploid cells. PI staining revealed an increase in the fractions of 8N in K562 cells (29.17% versus 16.83%), MEG-01 cells (28.72% versus 17.97%), and UT-7 cells (39.38% versus 15.2%) in the medium with SI compared to control. The ABI also had a profound effect on the percentage of ploidy formation (44.60%; 31.70%; 34.43%) in K562, MEG-01, and UT-7 cells, respectively (Figure 1(b)). To evaluate whether small molecules would affect cell survival in culture, we also analyzed cell proliferation. The small molecules did not induce significant cytotoxicity or downregulation of surviving in human leukemic cells (Figure 1(c)).

**Figure 2:**
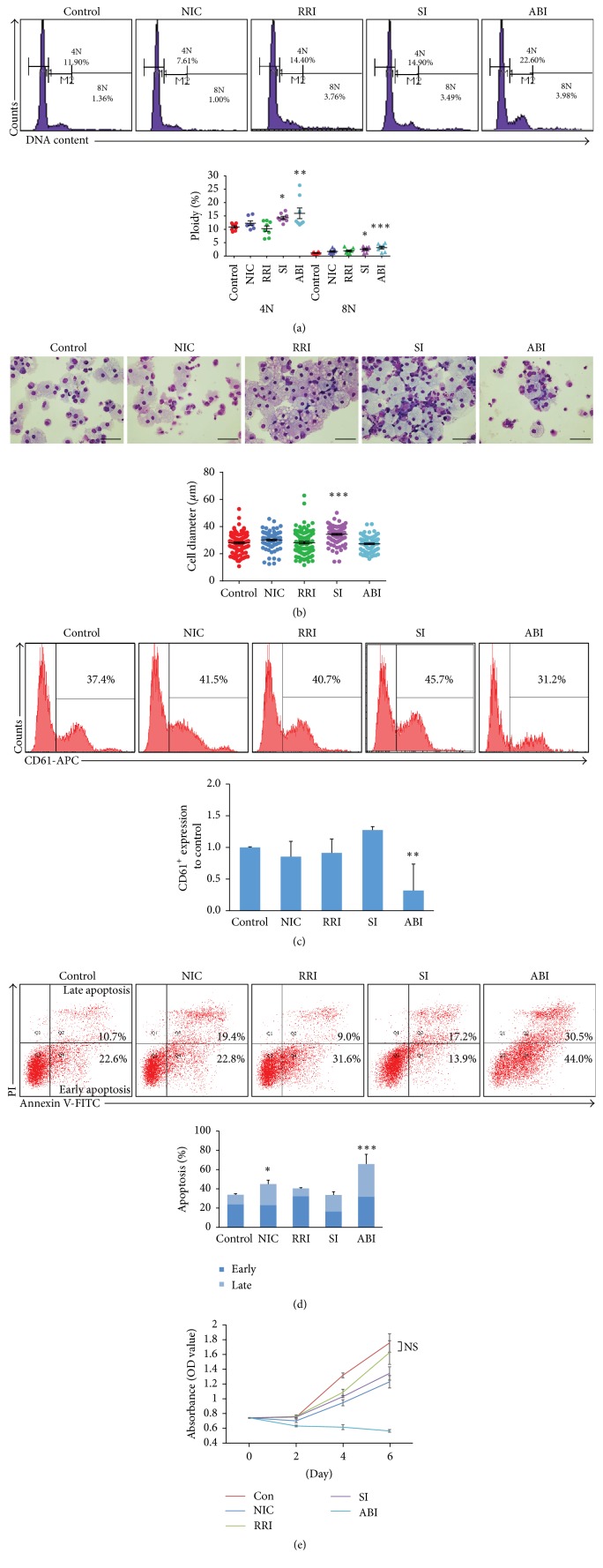
Effect of small molecules on MK polyploidization in human MK progenitors (MK-PROs). (a) MK-PROs DNA ploidy analysis (4N and ≥8N) after different small molecules treatment. Representative flow cytometry plots are shown in the upper panel. Statistical analysis was made by comparing with the control group. (b) Morphology and cell diameter of MK-PROs after small molecules treatment for 10 days (scale bars: 50 *μ*m). (c) The proportion of CD61^+^ cells after treated with four small molecules was normalized to control. (d) The apoptosis analysis by flow cytometry in MK-PROs. Histogram of the proportion of early apoptosis and late apoptosis in MK-PROs treated with or without small molecules is showed in the lower panel. (e) The cell proliferation with or without small molecules. Results are means and SEM from biological replicates (*n* = 8). ^*∗*^*p* < 0.05; ^*∗∗*^*p* < 0.01; ^*∗∗∗*^*p* < 0.001.

To make our result more convincing and reflect normal hematopoiesis, simultaneously, we examined the effects of small molecules on polyploidization in human MK progenitors (MK-PROs). Ploidy analysis by flow cytometry showed that the proportion of 4N cells and 8N cells by treated with SI (14.33%, 2.46%) or ABI (15.99%, 3.14%) was also significantly increased when compared with control (10.85%, 1.11%), which is consistent with previous observations. The proportion of 4N and 8N cells in NIC-treated cells increased but not significant. Besides, the proportion of 4N and 8N cells in RRI had no significant as well (Figure 2(a)). When evaluating megakaryocyte maturity by cellular morphology, the 8N polyploidy was observed in both SI-treated and ABI-treated MK-PROs. However, the MK-PROs underwent cell-size enlargement only could be found after SI treatment (Figure 2(b)). Additional, we analyzed the expression of the megakaryocytic marker. No significant difference in percentage of CD61 positive cells could be detected between the MK differentiation culture with or without the small molecules except for ABI, which reduced the percentage by 68% (Figure 2(c)). Furthermore, we observed ABI-treatment caused massive cell apoptosis and death. The Apoptosis Detection analysis was performed and the result showed that the ABI-treated cells exhibited great amount apoptosis (65.78%) and the NIC-treatment also caused cell apoptosis (44.95%) compare to control (33.88%) (Figure 2(d)). The cell amplification blockage resulting from ABI-treatment was confirmed by cell proliferation assay (Figure 2(e)). Together with cell cycle analysis, we found that ABI did not inhibit cell cycle but caused cell apoptosis, which is consistent with a previous report [8]. Taken together, SI and ABI could significantly improve the percentage of polyploidy both in leukemic cells and MK-PROs. However, the addition of ABI resulting in cell death and apoptosis excludes it from an efficient MK differentiation supplement and future clinical application. So, a formula supplemented with SI would be optimal.

### 3.2. The Combination of Small Molecules for MK Polyploidization

**Figure 3:**
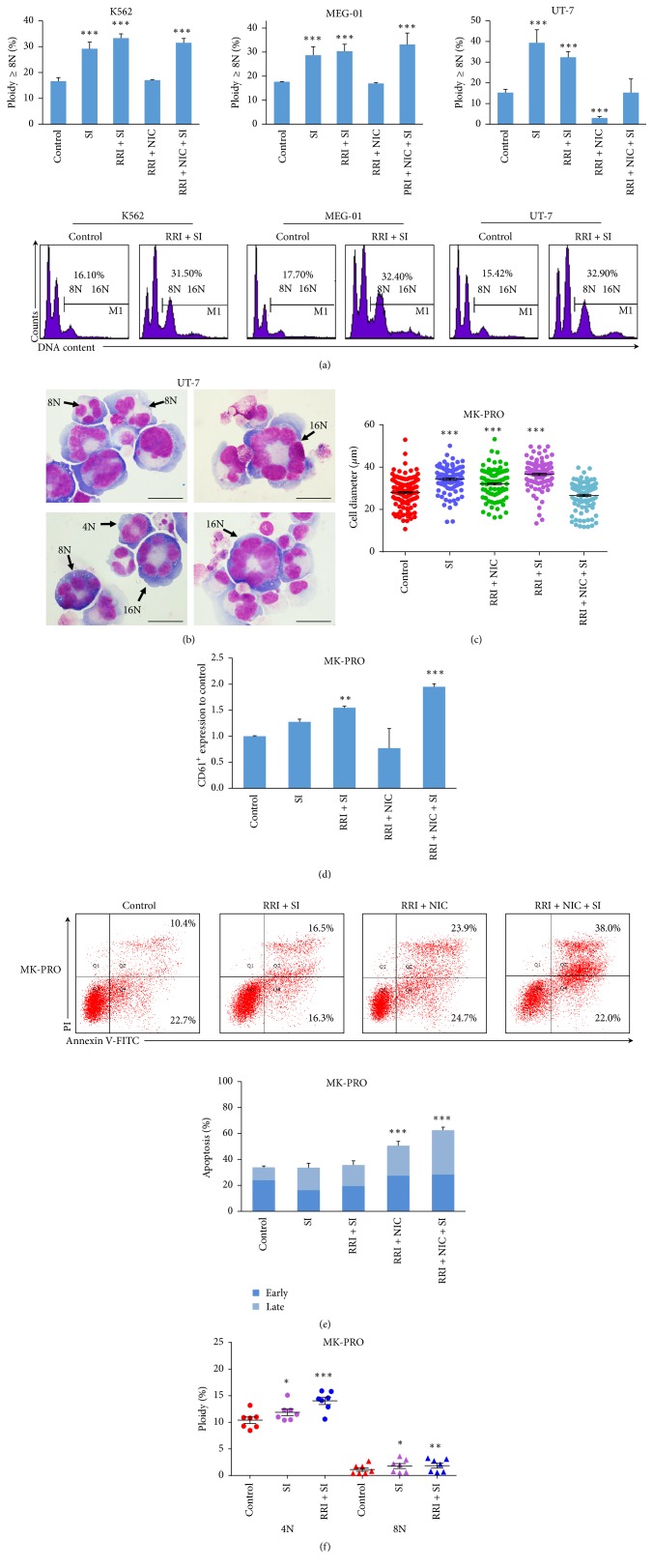
The combination of small molecules for polyploidization in MKs. (a) Ploidy analysis by flow cytometry. Ploidy (≥8N) after the combination of small molecule treatment was compared with control in three megakaryocytic cells. Representative flow cytometry plots of RRI + SI treatment or control are shown in the lower panel. (b) Morphology of stained polyploid megakaryocytes in UT-7 cells treated with RRI and SI (scale bars: 20 *μ*m). (c) Cell diameter of the MK-PROs after small molecule combinations treatment for 10 days. (d) The proportion of CD61^+^ cells from MK-PROs treated with the combined small molecules, normalized to control. (e) The early apoptosis and late apoptosis were detected by flow cytometry. Lower panel: the proportion of apoptosis in MK-PROs treated with combined small molecules, compared with control. (f) Ploidy (4N and ≥8N) in MK-PROs treated with RRI and SI was compared with control. Results are means and SEM from biological replicates (*n* = 7). ^*∗*^*p* < 0.05; ^*∗∗*^*p* < 0.01; ^*∗∗∗*^*p* < 0.001.

To gain a better promotion of polyploidization, we further examined the effect of different combinations of small molecules in cell lines and MK-PROs. In cell lines, our results indicated that the addition of SI alone or with other small molecules (SI, SI + RRI and SI + RRI + NIC group) would significantly enhance the percentage of ploidy in all cells besides the combination of RRI, SI, and NIC in UT-7 cells. We considered that it might be because of the inhibited effect of NIC according to the result from NIC-treated alone. Treatment with RRI and SI combination had additive effect for polyploidization compared with SI alone in K562 (33.30% versus 29.17%) and MEG-01 cells (30.35% versus 28.72%). The combination of NIC, RRI, and SI increased polyploid relative to control group but was no better than the combination of RRI and SI in both K562 and UT-7 cells (Figure 3(a)). After RRI and SI treatment, the cells showed ploidy characteristics with 8N, 16N, and even 32N in UT-7 cells. The high polyploidy cells were significantly larger compared to low ploidy (Figure 3(b)). The effect of these small molecule combinations was also examined in MK-PROs. MK-PROs treated with SI, SI, and RRI, RRI, and NIC showed more mature MK phenotypes, as larger cell size and higher polyploid than control (Figure 3(c)). RRI and SI combination and combinations of RRI, NIC, and SI treated MK-PROs showed higher CD61+ percentage compared to control (Figure 3(d)). Additional, RRI and SI treatment did not affect cell apoptosis. The number of MK-PROs in the groups with NIC (RRI + NIC and RRI + NIC + SI) was significantly decreased with notable apoptosis (Figure 3(e)). Therefore, we excluded NIC from the MK differentiation culture system and specifically investigated the effect of RRI and SI treatment on MK-PROs. The proportion of 4N and 8N cells by treatment with RRI and SI increased significantly and was better than SI alone (Figure 3(f)). Combination of RRI and SI could increase the expression of CD61 and enhanced the polyploid level without a statistically significant cell apoptosis, which indicated that RRI and SI were the optimal small molecule combinational supplement for MK polyploidization.

### 3.3. Effect of Small Molecules on Cell Cycle Regulators and Cytoskeletal Proteins

**Figure 4:**
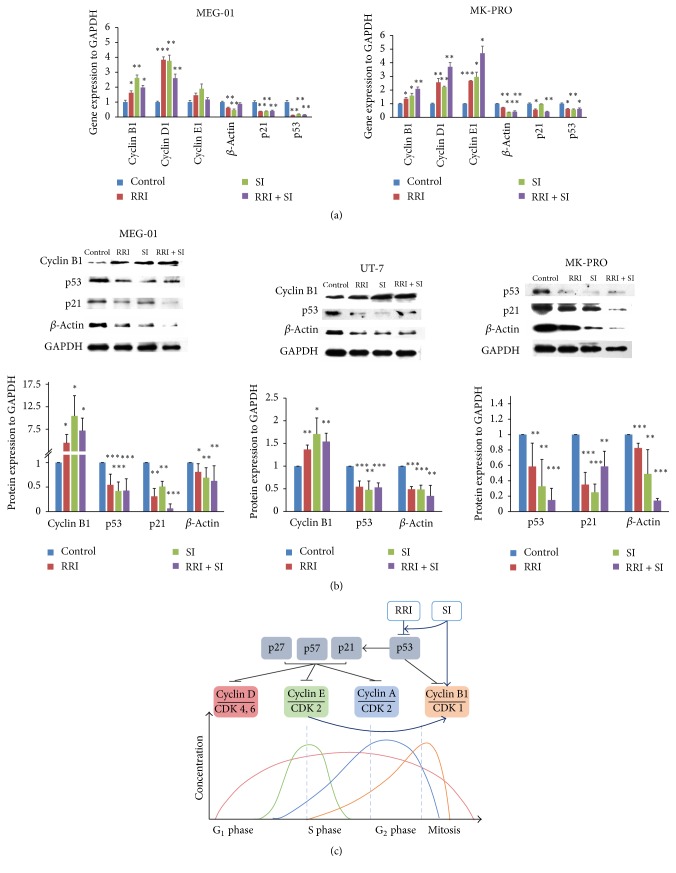
Expression of cell cycle-related proteins and cytoskeletal proteins after RRI and SI treatment. (a) Expression of cyclin B1, cyclin D1 cyclin E1, *β*-actin, p21, and p53 detected by Q-PCR in MEG-01 cells and MK-PROs. (b) The p53, p21, *β*-actin, and cyclin B1 expression detected by western blot in MEG-01, UT-7, and MK-PROs. The intensity of each band was digitalized and compared by Image Lab software. (c) Schematic diagram of the relationship of small molecules and cell cycle-related proteins. ^*∗*^*p* < 0.05; ^*∗∗*^*p* < 0.01; ^*∗∗∗*^*p* < 0.001.

In megakaryocyte polyploidization, the cell cycle-related protein and cytoskeletal proteins both play important role in MK endomitosis. It is suggested that G1 phase progression protein cyclin D1 and G1/S checkpoint protein cyclin E1 are both essential for DNA content enlargement and megakaryocyte polyploidy. Moreover, the maintenance of M phase protein cyclin B1 is required for G2 phase arresting and megakaryocyte endomitosis. As a consequence, the expression of cell cycle regulator cyclin D1, cyclin E1, and cyclin B1 under small molecule treatment was checked first to uncover the function of small molecules. Cyclin B1, cyclin D1, and cyclin E1 increased significantly by RRI and/or SI treatment in leukemic cells and MK-PROs by mRNA level. The additive effect of RRI and SI on cyclins expression was more profound in MK-PROs. Actin inhibition which might abrogate the actin cleavage furrow formation has been proved essential in the megakaryocyte polyploidization. It was also clear in our study that the expression of *β*-actin normalized to GAPDH reduced significantly in all cells treated with RRI and SI (Figure 4(a)).

To figure out the direct connection between small molecules and cyclin regulation, key regulators of cyclin-CDK complex, CDK inhibitors (CKIs), and their upstream molecules were explored. Hints from the studies on RRI and SI in cell cycle regulating suggest that a crucial cell cycle regulator, p53, can be suppressed both by the two small molecules [9, 10]. A direct target of p53, also a pivotal CKI, p21^WAF1/CIP1^ [11], can suppress the activation of both cyclin D-CDK4/6 and cyclin E-CDK2 complexes [12–15]. It has also been found that p53 prevents G2/M transition by decreasing intracellular levels of cyclin B1 protein and attenuating the activity of the cyclin B1 promoter [16]. Accordingly, we narrowed our analysis of potential RRI and SI targets in endomitosis to cell cycle regulators, especially to p53, p21, cyclinB1, and *β*-actin. The western blot demonstrated a decrease in p53, p21 and *β*-actin expression and an increase in cyclin B1 expression in response to RRI and SI treatment (Figure 4(b)). Therefore, besides direct downregulation of the level of actin protein, RRI and SI reduce the level of p53 and p21, subsequently invert their inhibition on cyclin D or cyclin E, probably together with a direct promotion of cyclin B with SI, and finally result in MK endomitosis.

## 4. Discussion

For a better platelet manufacture, the small molecules such as NIC, RRI, SI, and ABI have all been reported had positive effect on MK polyploidization [7, 17]. However, the best cell culture supplement or supplement combination from these small molecules is still controversial. We took advantage of MK differentiation model to optimize small molecule supplements for MK polyploidization. A summary of the impact of the small molecules is provided in Table 2 and it clearly shows that ABI had the most effect in inducing ploidy in K562, MEG-01 and UT-7 cells when the small molecules were added, respectively. However, the cells were strongly apoptosis after ABI treatment, which prevented us from taking ABI as suitable supplement for MK induction.

In K562, MEG-01, and UT-7 cells, RRI had no effect for ploidy, which was different from previous report [7]. The effect of RRI on primary megakaryocyte polyploidization was detectable but not dramatic. Considering the great individual variation between each cord blood samples, one would expect that studies coming from cell lines are more reliable and replicable. The treatment with SI resulted in significant augment of polyploidization in all 3 cell lines. Moreover, when combined RRI with SI, the percentage of ploidy was even higher than SI treating alone. In the viewpoint of cytokinesis regulation, RRI is an inhibitor of RhoA signal pathway, which influences contractile ring formation and correctly completed cytokinesis [18]. While SI presents Src family tyrosine kinase inhibitor SU-6656, it inhibits actin polymerization and prevents cell from cytokinesis [19]. There might be cross-talk between Rock and Src signal pathways; as a consequence, by cosuppression of Rock and Src pathways with RRI and SI, the lever of ploidy significantly increased.

Although RRI, SI, and ABI presented common effect on all the three cell lines we tested, treatment of NIC inhibited the polyploidization of UT-7 cells instead of showing no effect. We suspect that the UT-7 cells used in this study had been pretreated with EPO, an erythroid lineage inducer, and an antiapoptosis factor as well, while NIC could increase the expression of p53 and cause apoptosis [20], and the megakaryocytic induction system without EPO made UT-7 cell more sensitive to NIC treatment.

In the mechanism studies, although initially the 4 small molecules were chosen based on their profound effect on cytokinesis, they have also been proved playing important roles in cell cycle regulation [9, 21–25]. Considering the importance of both DNA replication and cytokinesis blockage on megakaryocyte polyploidization, effect of the small molecules on cell cycle regulators and cytoskeleton was examined side by side. Cyclin D1 is a G1 phase cyclin, and it was used to be elevated for DNA synthesis. Its overexpression results in enlarged megakaryocyte size and increased DNA content [26]. Cyclin E/CDK2 complex is required for G1/S phase transition, and it is also essential for megakaryocyte polyploidy [27]. Besides, cyclin B1 expression could be elevated by cyclin E upregulation. And a sustained expression of cyclin B1 was also observed in polyploid cells [27, 28]. As expected, the expression of cyclin D1, cyclin E, and cyclin B1 elevated together with the improved ploidy after SI or RRI and SI treatment. Studies of the upstream signal of cyclins also revealed that p53 and p21 are the key connectors between SI/RRI treatment and cyclin upregulation. Both SI and RRI function on the suppression of p53 and its target p21; the latter serves as an inhibitor of G1/S cyclin-CDK complexes [9, 10, 21]. And the expression of cyclin B1 could be directly upregulated by SI as previously reported [24]. Taken together, the small molecules suppress the expression of p53 and p21, promote the level of cyclin D, cyclin E, and cyclin B, and thus enhance DNA content and endomitosis, together with cytokinesis blockage through actin downregulation, results in the promotion of megakaryocyte polyploidy (Figure 4(c)).

In conclusion, by using leukemia cell line megakaryocytic induction model, a small molecule combination was discovered as polyploidization promoter. The function of the combination was proved through downstream signal examination. Hopefully, the small molecules might contribute to in vitro platelet manufacture in a sooner future.

## Supplementary Material

Supplementary results on megakaryocyte inuction.

## Figures and Tables

**Table 1:** Primer sequence.

Gene	Primer sequence (5′-3′)	Annealing temperature (°C)
Cyclin B1	F: AATAAGGCGAAGATCAACATGGC	58
	R: TTTGTTACCAATGTCCCCAAGAG	
Cyclin D1	F: GCTGCGAAGTGGAAACCATC	58
	R: CCTCCTTCTGCACACATTTGAA	
Cyclin E1	F: GCCAGCCTTGGGACAATAATG	58
	R: CTTGCACGTTGAGTTTGGGT	
p21	F: TGTCCGTCAGAACCCATGC	58
	R: AAAGTCGAAGTTCCATCGCTC	
p53	F: CCCCTCCTGGCCCCTGTCATCTTC	58
	R: GCAGCGCCTCACAACCTCCGTCAT	
*β*-Actin	F: CATGTACGTTGCTATCCAGGC	58
	R: CTCCTTAATGTCACGCACGAT	
GAPDH	F: GAGTCAACGGATTTGGTCGT	58
	R: TTGATTTTGGAGGGATCTCG	

**Table 2:** Impact of four small molecules and their combinations on megakaryocyte maturation judged by K562, UT-7, MEG-01 cells, and primary MK progenitors (MK-PROs).

	NIC	RRI	SI	ABI	RRI + SI	RRI + NIC	RRI + SI + NIC
Cell diameter	—	↑^1^	↑	↑^2^	↑	↑	↑^3^
DNA ploidy	—^4^	—	↑	↑	↑	—^5^	↑
Proliferation	—	—	—	—^6^			
CD61 expression^7^	—	—	—	↓	↑	—	↑
Apoptosis^7^	↑	—	—	↑	—	↑	↑

^1,2^RRI or ABI did not increase the cell diameter of MK-PROs; ^3^RRI + SI + NIC did not increase the cell diameter of MK-PROs and UT-7 cells; ^4,5^NIC alone or with SI reduced the percentage of highly polyploid cells in UT-7 cells; ^6^ABI-treatment resulted in cell amplification blockage in MK-PROs; ^7^CD61 expression and apoptosis were summarized from the result of MK-PROs.
