# Supplementary material for: Small Molecule Supplements Improve Cultured Megakaryocyte Polyploidization by Modulating Multiple Cell Cycle Regulators
Source: Biomed Res Int. 2017 Oct 19;2017:2320519. doi: 10.1155/2017/2320519 (PMC5671672; doi:10.1155/2017/2320519)
Supplement: Supplementary file 1 — Supplementary results on megakaryocyte inuction. [file 2320519.f1.pdf]

Supplementary materials

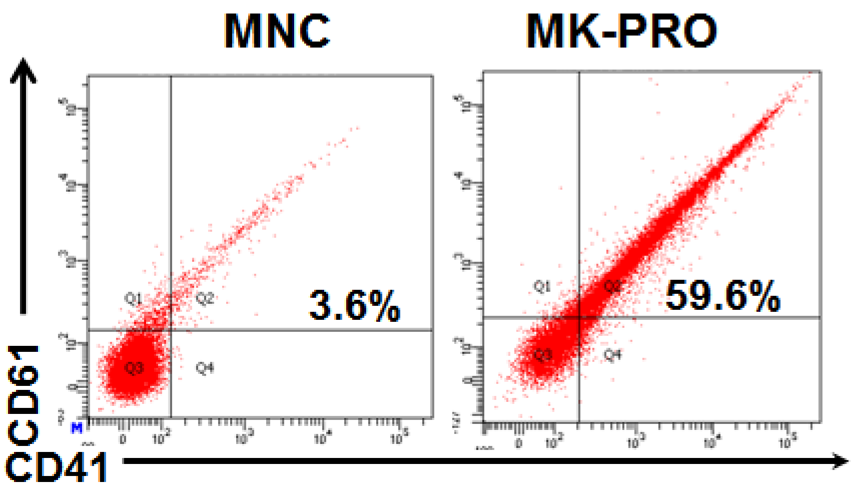

Figure S1. Representative flow cytometric plots of human umbilical cord blood and the MK progenitors after 10 days culture.

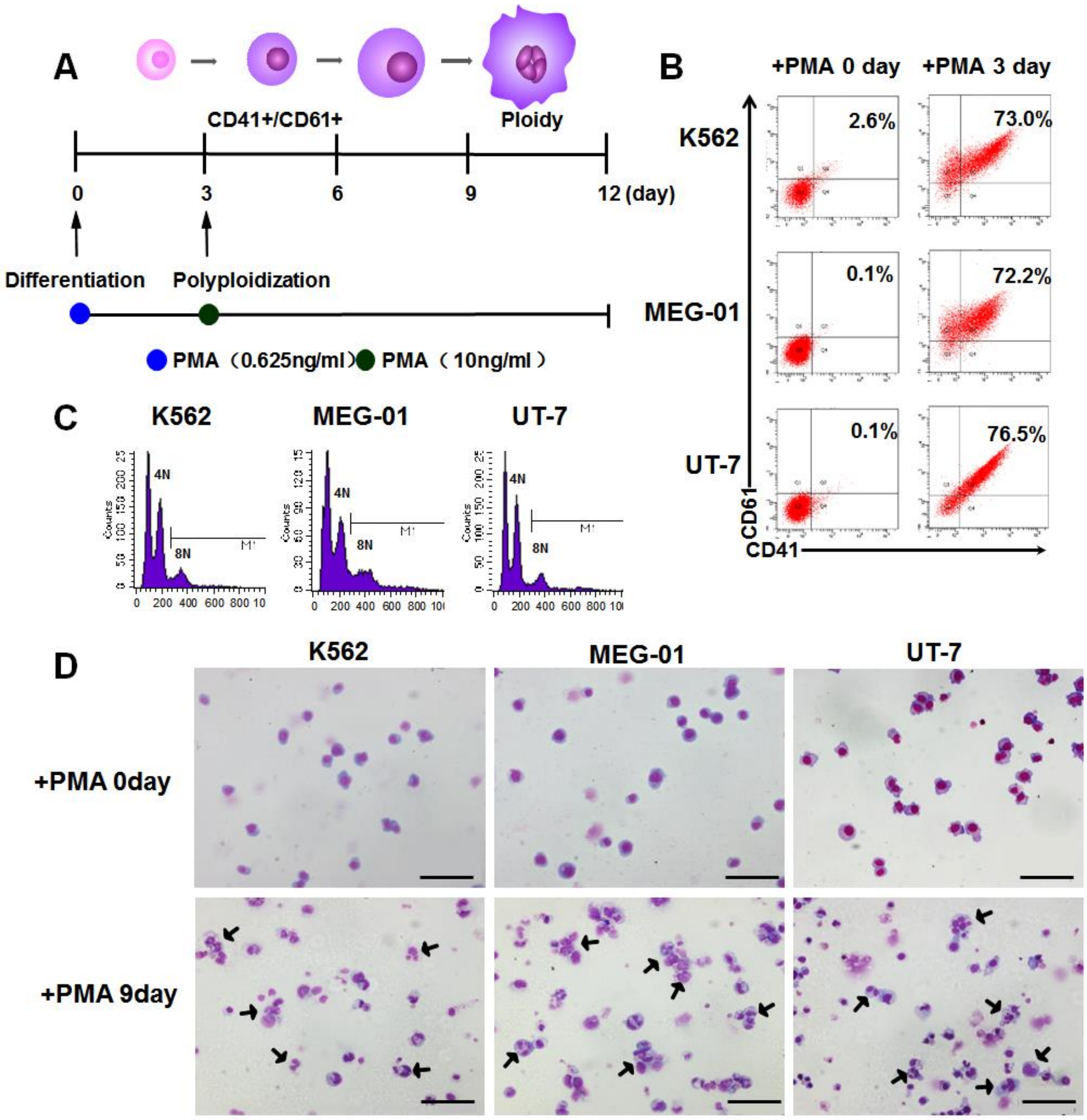

Figure S2.Optimization of the culture method for megakaryocyte polyploidization in leukemic cell lines.

(A) Schematic protocols of step-wise polyploidization induction in cell lines with PMA. (B) The proportion of CD41^{+}/CD61^{+} cells in K562, MEG-01 and UT-7 cells before and after induction. (C) DNA ploidy analysis by flow cytometry in K562, MEG-01 and UT-7 cells. (D) Morphology of three kinds of cells before and after PMA treatment. Cells were stained with Wright-Giemsa solutions. (Scale bars: 100 μm)

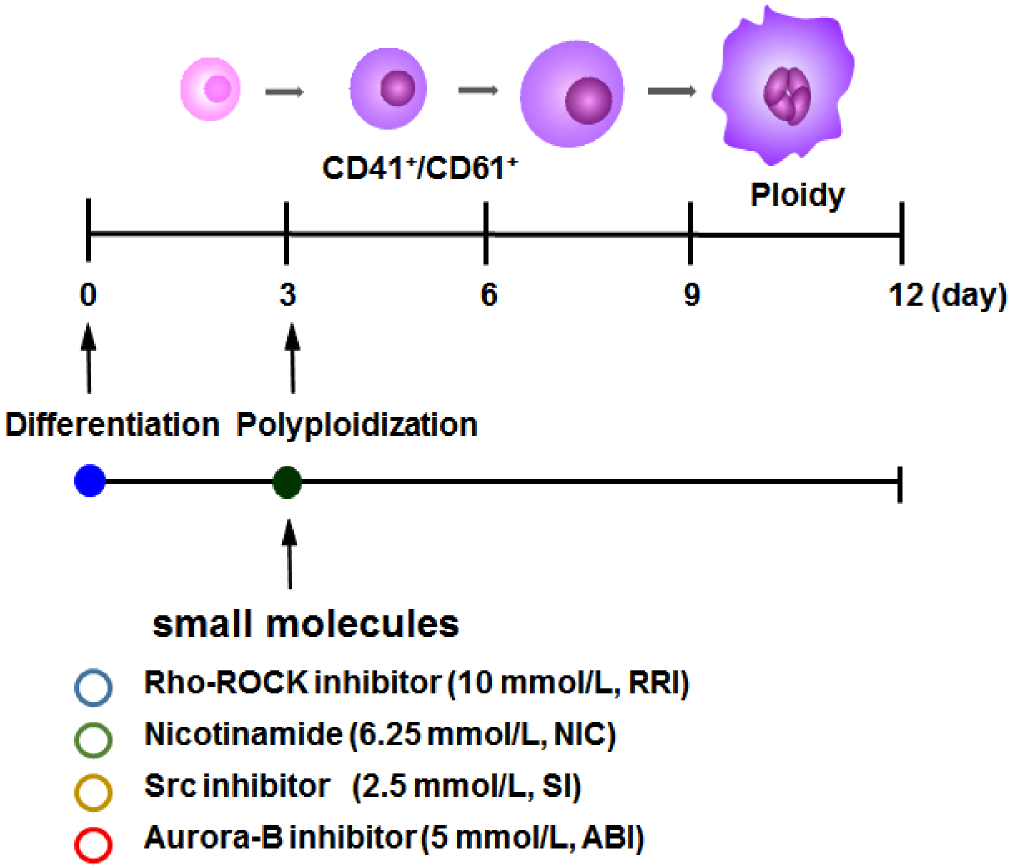

Figure S3. Schematic protocols of step-wise polyploidization induction in cell lines with different small molecules addition.
